# Prevalence of biliary gastritis and associated demographic, dietary, and clinical factors among adults in the Kurdistan Region of Iraq: a cross-sectional study

**DOI:** 10.1186/s13104-026-07854-y

**Published:** 2026-05-07

**Authors:** Araz Omar Fatah, Kochr Ali Mahmood, Dawan Jamal Hawezy, Dilshad Hamad Mustafa, Saman Taher Barzinjy, Sirwan Khalid Ahmed

**Affiliations:** 1 Faculty of Medicine, Koya University, Koya, Kurdistan Region Iraq; 2 College of Medicine, Hawler Medical University, Erbil, Kurdistan Region Iraq; 3 College of Nursing, University of Raparin, Rania, Sulaymaniyah, Kurdistan Region 46012 Iraq

**Keywords:** Biliary gastritis, Bile reflux, Duodenogastric reflux, Diet, Gallstones, Kurdistan Region

## Abstract

**Objective:**

Biliary gastritis is an under-recognized inflammatory condition associated with duodenogastric bile reflux and nonspecific gastrointestinal symptoms, often leading to diagnostic challenges. Epidemiological data from the Kurdistan Region of Iraq are limited. This study aimed to estimate the prevalence of biliary gastritis among adults with available diagnostic data and to examine its associations with demographic, lifestyle, dietary, and clinical factors.

**Results:**

A descriptive cross-sectional study was conducted between June 2024 and April 2025 among 638 adults recruited from urban and rural healthcare centers. Biliary gastritis was identified based on documented clinical diagnosis and/or prior endoscopic findings. Among participants with available diagnostic documentation (*n* = 486), the prevalence of biliary gastritis was 26.7%. The mean age of participants was 43.53 ± 15.25 years. Significant associations were observed with sex, marital status, occupation, dietary factors (fast food consumption, fruit and vegetable intake, and caffeine intake), gallstone disease, liver disease, and gastrointestinal symptoms including nausea, vomiting, and abdominal pain (*p* < 0.05). No significant associations were found with diabetes mellitus, gastroesophageal reflux disease, or physical activity. Multivariable logistic regression identified several demographic, dietary, and clinical variables associated with biliary gastritis. These findings suggest that biliary gastritis represents a notable health concern in this setting, highlighting the importance of dietary modification and improved access to diagnostic services. However, findings should be interpreted cautiously due to the cross-sectional design and reliance on facility-based data. Further longitudinal studies are warranted.

## Introduction

Biliary gastritis is an inflammatory condition associated with duodenogastric bile reflux, which affects the gastric mucosa through prolonged exposure to bile acids and duodenal contents. It is frequently under-recognized, as its clinical manifestations often overlap with other gastrointestinal disorders, leading to delayed diagnosis or misclassification [1, 2].

The pathophysiology of biliary gastritis involves disruption of the gastric mucosal barrier secondary to bile reflux, which may arise from biliary obstruction, impaired gastric or pyloric motility, or postoperative anatomical alterations such as cholecystectomy or gastric surgery [3].

Diets high in fat and low in dietary fiber have been associated with biliary disorders through altered bile composition and impaired gastric motility [4]. In the Kurdistan Region, traditional dietary patterns and ongoing lifestyle transitions may contribute to an increased burden of biliary disease [5].

Healthcare access disparities may further complicate disease detection and management. In rural areas of the Kurdistan Region, restricted availability of endoscopic services may lead to underdiagnosis or delayed recognition of biliary gastritis [6]. Chronic, untreated bile reflux has been associated with mucosal injury and long-term complications, including peptic ulcer disease and gastric atrophy [7].

Cultural practices and healthcare-seeking behaviors may also influence disease presentation. Delayed medical consultation and reliance on traditional remedies may exacerbate symptom persistence and hinder timely diagnosis. Additionally, *Helicobacter pylori* infection—common in the Kurdistan Region—may coexist with biliary gastritis, further complicating clinical assessment [8, 9].

Despite growing global interest, epidemiological data on biliary gastritis from the Kurdistan Region remains limited. Accordingly, this study aimed to estimate the prevalence of biliary gastritis among adults with available diagnostic data and to examine demographic, lifestyle, dietary, and clinical factors associated with the condition in the Kurdistan Region of Iraq.

## Methods

### Study design and setting

This study employed a descriptive cross-sectional design to estimate the prevalence of biliary gastritis among adults with available diagnostic data and to examine demographic, lifestyle, dietary, and clinical factors associated with the condition among adults in the Kurdistan Region of Iraq. Data were collected between 21 June 2024 and 15 April 2025 from selected healthcare centers located in both urban and rural areas to reflect routine healthcare access and enhance representativeness across population settings.

### Study population

The study population comprised adults aged 18 years and older who were residents of the Kurdistan Region at the time of data collection and who attended the selected healthcare facilities. Individuals who provided informed consent and met the eligibility criteria were enrolled. Participants with a history of total gastrectomy or major gastric reconstructive surgery were excluded because these procedures substantially alter bile reflux physiology. Individuals who declined to participate or were unable to provide informed consent were also excluded.

### Sample size and sampling technique

A total of 638 participants were included, based on feasibility and availability of eligible attendees during the study period. A multistage sampling approach was employed to enhance representativeness across the Kurdistan Region. Initially, major urban centers and rural districts from different governorates were purposively identified to capture geographic diversity. Subsequently, healthcare facilities within these areas were randomly selected using a sampling frame of eligible centers. Within each selected facility, participants were recruited using simple random sampling from eligible adult attendees during the study period. This approach aimed to minimize selection bias and improve the generalizability of findings across urban–rural populations and healthcare access levels. However, as the study was facility-based, findings may not fully represent individuals who do not seek healthcare services.

### Data collection tools and procedures

Data were collected through face-to-face interviews using a structured questionnaire developed by the research team based on a review of relevant literature and prior studies [10–12]. The questionnaire was piloted for clarity and feasibility prior to data collection and was administered uniformly by trained data collectors.

The questionnaire included the following domains:

- Sociodemographic characteristics: age, sex, place of residence, marital status, and occupation.
- Lifestyle factors: smoking status, alcohol consumption, and physical activity.
- Dietary habits: frequency of fast-food consumption, spicy food intake, caffeine consumption, and fruit and vegetable intake.
- Gastrointestinal symptoms: nausea, vomiting, abdominal pain, heartburn, and bloating.
- Healthcare utilization and family history of gastrointestinal or biliary disease.

Anthropometric measurements (height and weight) were recorded when available to assess obesity status. Clinical history—including gallstones, liver disease, diabetes mellitus, medication use, and previous surgical procedures—was obtained through participant self-report and verified using medical records whenever accessible to reduce misclassification.

Endoscopic findings, including gastric erythema, bile pooling, ulcers, or erosions, were documented only for participants who had previously undergone upper gastrointestinal endoscopy as part of routine clinical care. No additional endoscopic or invasive diagnostic procedures were performed specifically for research purposes, reflecting real-world diagnostic practice in the region.

### Outcome definition

Biliary gastritis was defined as (a) a documented clinician diagnosis of bile reflux gastritis or biliary gastritis recorded in medical files and/or (b) endoscopic findings consistent with bile reflux gastritis (e.g., visible bile reflux in the stomach accompanied by erythematous or erosive gastric mucosa), where endoscopy reports were available. Participants without clinical documentation or endoscopic evidence were coded as having missing outcome data rather than being classified as disease-free, to minimize misclassification and verification bias in prevalence estimation.

### Ethical considerations

The study was conducted in accordance with the ethical principles of the Declaration of Helsinki. Ethical approval was obtained from the Ethics Committee of the Faculty of Medicine, Koya University (Meeting Code: 19; Paper Code: 17). All participants were informed about the study objectives, procedures, and their rights prior to participation. Oral informed consent was obtained in line with institutional ethical guidance. Confidentiality and anonymity were strictly maintained, and participation was voluntary, with the right to withdraw at any stage without consequences.

### Data analysis

Descriptive statistics—including frequencies, percentages, means, and standard deviations—were used to summarize participant characteristics. Associations between biliary gastritis and demographic, lifestyle, dietary, and clinical variables were initially assessed using chi-square tests. Variables demonstrating statistical significance in bivariate analysis or considered clinically relevant a priori were entered into multivariable logistic regression models to estimate odds ratios (ORs) with 95% confidence intervals. Results were interpreted as associations rather than causal effects, consistent with the cross-sectional study design. A p-value of ≤ 0.05 was considered statistically significant. Variables with sparse cell counts (e.g., alcohol use) were interpreted cautiously due to limited variability in exposure. All statistical analyses were conducted using SPSS version 27.

## Results

### Participant characteristics (revised)

A total of 638 participants were included, with effective sample sizes varying across analyses due to missing diagnostic or variable-specific data. Demographic and lifestyle characteristics are summarized in Table 1. Participants were predominantly married and middle-aged, with a slight male predominance and a near-equal urban–rural distribution. Approximately half were employed, most reported no alcohol use and were non-smokers, and fewer than one-third engaged in regular physical activity. The mean age was 43.53 ± 15.25 years.

**Table 1:** Frequency and percentage distributions of demographic and lifestyle characteristics of the participants (*N* = 638)

Variable	Category	Frequency (*n*)	Percent (%)
Gender	Male	358	56.1
	Female	280	43.9
Marital status	Single	138	21.6
	Married	500	78.4
Residence	Urban	334	52.4
	Rural	304	47.6
Occupation	Employed	336	52.7
	Unemployed	284	44.5
	Student	18	2.8
Smoking status	No	442	69.3
	Yes	196	30.7
Alcohol use	No	606	95
	Yes	32	5
Physical activity	None	468	73.4
	1–2 times/week	170	26.6
Mean of age ± S.D	43.53 ± 15.25		

### Dietary habits

Dietary habits of participants are summarized in Table 2. Low intake of fruits and vegetables was common, with fewer than one-quarter of participants reporting consumption more than once weekly. Fast food consumption was reported by approximately one-third of participants, while most participants reported limited intake of spicy foods. Daily caffeine consumption was uncommon, with only a small proportion consuming caffeine one or more times per day.

**Table 2:** Frequency and percentage distributions of dietary habits among participants (*N* = 638)

Variable	Category	Frequency (*n*)	Percent (%)
Spicy food consumption	Never	400	62.7
	Rarely	238	37.3
Fast food consumption	No	414	64.9
	Yes	224	35.1
Fruit and vegetable intake	Rarely	368	57.7
	Rarely/once a week	192	30.1
	3–4 times per week	64	10
	Daily	14	2.2
Caffeine intake	Rarely	340	53.3
	Occasionally	270	42.3
	1–2 times per day	28	4.4

### Clinical characteristics and gastrointestinal conditions

The distribution of gastrointestinal and related medical conditions is presented in Table 3. Gallstone disease and peptic ulcer disease were among the more frequently reported conditions, whereas gastroesophageal reflux disease, liver disease, and diabetes mellitus were less common. More than half of participants reported current medication use, reflecting the burden of chronic or recurrent gastrointestinal symptoms within the study population.

**Table 3:** Distribution of gastrointestinal conditions among participants (*N* = 638)

Variable	Category	Frequency (*n*)	Percent (%)
Gastroesophageal reflux disease (GERD)	No	578	90.6
	Yes	60	9.4
Peptic ulcer disease	No	350	54.9
	Yes	288	45.1
Gallstones	No	468	73.4
	Yes	170	26.6
Liver disease	No	564	88.4
	Yes	74	11.6
Diabetes	No	592	92.8
	Yes	46	7.2
Taking medication	No	226	35.4
	Yes	412	64.6

### Gastrointestinal symptoms

Gastrointestinal symptoms reported by participants are summarized in Table 4. Abdominal pain was the most commonly reported symptom, followed by nausea and vomiting, heartburn, and bloating. Most symptomatic participants reported symptom duration of less than six months, suggesting relatively recent or intermittent symptom onset in the majority of cases.

**Table 4:** Distribution of symptoms related to gastritis (*N* = 638)

Variable	Category	Frequency (*n*)	Percent (%)
Nausea and vomiting	No	538	84.3
	Yes	100	15.7
Heartburn	No	522	81.8
	Yes	116	18.2
Stomach pain	No	376	58.9
	Yes	262	41.1
Bloating	No	542	85
	Yes	96	15
Loss of appetite	No	574	90
	Yes	64	10
Duration of symptoms	< 1 Month	308	48.3
	1–6 Months	248	38.9
	6 Months – 1 Year	82	12.9

### Prevalence of biliary gastritis

**Fig. 1:**
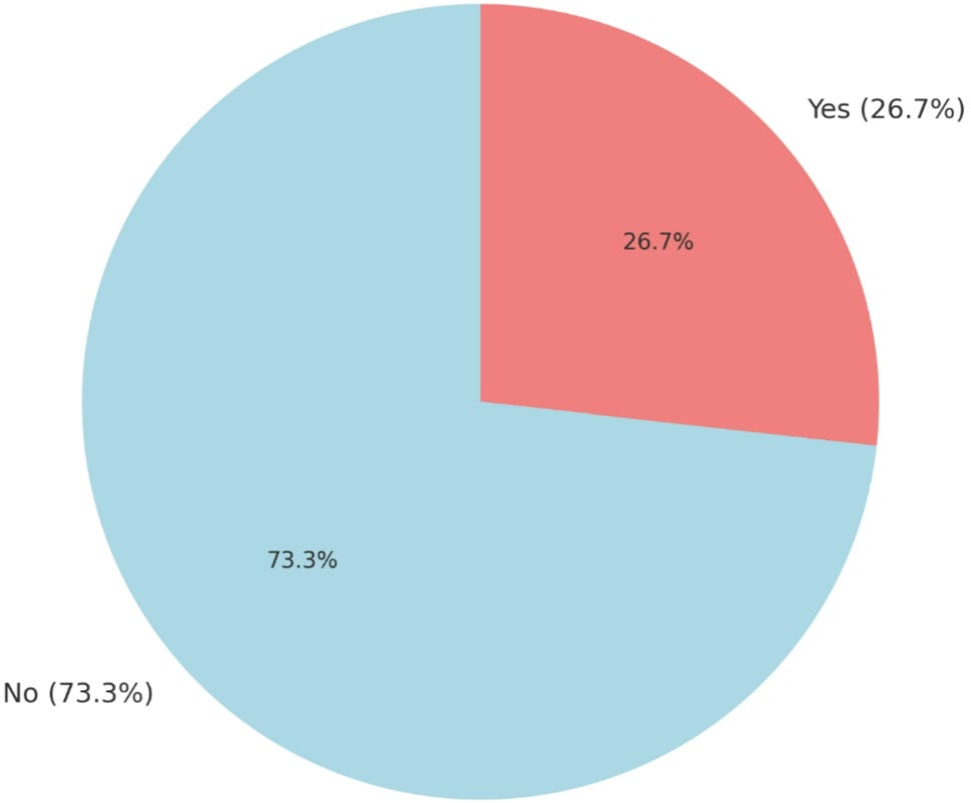
Distribution of biliary gastritis

The prevalence of biliary gastritis is illustrated in Fig. 1. Among participants with available diagnostic documentation (*n* = 486), 26.7% were diagnosed with biliary gastritis, while the remainder had no documented clinical or endoscopic evidence of the condition. Participants without diagnostic records were not classified as disease-free and were excluded from prevalence estimation, to minimize misclassification bias. Accordingly, prevalence estimates should be interpreted as representative of adults with available diagnostic documentation rather than the entire study sample.

### Healthcare utilization and endoscopic findings

Healthcare utilization patterns and diagnostic procedures are summarized in Table 5. Most participants reported infrequent or annual gastrointestinal healthcare visits, while only a minority had undergone endoscopic evaluation. A notable proportion reported a family history of gastric disease and previous episodes of melena. Among participants who underwent endoscopy as part of routine clinical care, gastric ulcers and erosions were the most reported findings.

**Table 5:** Distribution of healthcare visits, diagnostic testing, and endoscopic findings among the participants (*N* = 638)

Variable	Category	Frequency (*n*)	Percent (%)
Frequency of GI healthcare visits	Rarely	242	37.9
	Once a year	328	51.4
	2–3 times per year	50	7.8
	More than 3 times per year	18	2.8
History of endoscopy/colonoscopy	No	546	85.6
	Yes	92	14.4
Family history of gastric disease	No	482	75.5
	Yes	156	24.5
History of melena (dark stool)	No	394	61.8
	Yes	244	38.2
Ulcer findings	No	536	84
	Yes	102	16
Erosion findings	No	494	77.4
	Yes	144	22.6

### Associations with biliary gastritis

Table 6 presents bivariate associations between demographic and lifestyle variables and biliary gastritis diagnosis among participants with available diagnostic data (*N* = 624). A significantly higher proportion of biliary gastritis was observed among female participants compared with males (*p* = 0.004). Marital status was also significantly associated, with higher prevalence among married participants (*p* < 0.001).

**Table 6:** Association between demographic and lifestyle variables and diagnosed biliary gastritis (*N* = 624)

Variable	Diagnosed no (*n*, %)	Diagnosed yes (*n*, %)	*p* value
Gender			
Male	266 (77.3%)	78 (22.7%)	0.004
Female	188 (67.1%)	92 (32.9%)	
Marital status			
Single	124 (89.9%)	14 (10.1%)	< 0.001
Married	330 (67.9%)	156 (32.1%)	
Residence			
Urban	238 (71.3%)	96 (28.7%)	0.367
Rural	216 (74.5%)	74 (25.5%)	
Occupation			
Employed	258 (80.1%)	64 (19.9%)	< 0.001
Unemployed	178 (62.7%)	106 (37.3%)	
Student	18 (100.0%)	0 (0.0%)	
Smoking status			
No	304 (68.8%)	138 (31.2%)	0.001
Yes	150 (82.4%)	32 (17.6%)	
Alcohol use			
No	422 (71.3%)	170 (28.7%)	< 0.001
Yes	32 (100.0%)	0 (0.0%)	
Physical activity			
None	330 (72.7%)	124 (27.3%)	0.949
1–2/week	124 (72.9%)	46 (27.1%)	

Occupation demonstrated a significant association, with unemployed participants showing higher prevalence compared with employed participants, while no diagnosed cases were observed among students (*p* < 0.001). Smoking status showed a significant inverse association in unadjusted analysis, with non-smokers exhibiting higher prevalence than smokers (*p* = 0.001). Alcohol use was similarly inversely associated, as no diagnosed cases were observed among participants reporting alcohol consumption (*p* < 0.001). These findings should be interpreted cautiously given the small number of alcohol-consuming participants and students, which limited variability.

In contrast, place of residence (urban versus rural) and physical activity level were not significantly associated with biliary gastritis in bivariate analysis (*p* > 0.05).

### Dietary, clinical, and symptom associations

As shown in Table 7, biliary gastritis was significantly associated with dietary factors including fast food consumption, fruit and vegetable intake, and caffeine intake (*p* < 0.05). Significant clinical associations were observed with gallstone disease, liver disease, medication use, and gastrointestinal symptoms, particularly nausea/vomiting and abdominal pain (*p* < 0.05). Healthcare utilization frequency was also associated, with higher prevalence among participants reporting annual or more frequent gastrointestinal visits. In contrast, no significant associations were identified with spicy food intake, gastroesophageal reflux disease, diabetes mellitus, peptic ulcer disease, heartburn, bloating, loss of appetite, or family history of gastric disease.

**Table 7:** Association between dietary habits, clinical factors, and diagnosed biliary gastritis (*N* = 624)

Variable	Diagnosed no (*n*, %)	Diagnosed yes (*n*, %)	*p* value
Spicy food			
Never	276 (71.5%)	110 (28.5%)	0.370
Rarely	178 (74.8%)	60 (25.2%)	
Fast food			
No	276 (69.0%)	124 (31.0%)	0.005
Yes	178 (79.5%)	46 (20.5%)	
Fruit intake			
Rarely	248 (70.1%)	106 (29.9%)	< 0.001
Once a week	160 (83.3%)	32 (16.7%)	
3–4 times a week	32 (50.0%)	32 (50.0%)	
Daily	14 (100.0%)	0 (0.0%)	
Caffeine intake			
Rarely	234 (71.8%)	92 (28.2%)	0.01
Occasionally	206 (76.3%)	64 (23.7%)	
1–2 times a day	14 (50.0%)	14 (50.0%)	
Gastroesophageal reflux disease			
No	408 (72.3%)	156 (27.7%)	0.474
Yes	46 (76.7%)	14 (23.3%)	
Peptic ulcer disease			
No	248 (73.8%)	88 (26.2%)	0.523
Yes	206 (71.5%)	82 (28.5%)	
Gallstone			
No	312 (68.7%)	142 (31.3%)	< 0.001
Yes	142 (83.5%)	28 (16.5%)	
Liver disease			
No	426 (75.5%)	138 (24.5%)	< 0.001
Yes	28 (46.7%)	32 (53.3%)	
Diabetes			
No	422 (73.0%)	156 (27.0%)	0.613
Yes	32 (69.6%)	14 (30.4%)	
Using medication			
No	198 (93.4%)	14 (6.6%)	< 0.001
Yes	256 (62.1%)	156 (37.9%)	
Nausea and vomiting			
No	404 (77.1%)	120 (22.9%)	< 0.001
Yes	50 (50.0%)	50 (50.0%)	
Heartburn			
No	370 (72.8%)	138 (27.2%)	0.927
Yes	84 (72.4%)	32 (27.6%)	
Stomach pain			
No	252 (69.6%)	110 (30.4%)	0.038
Yes	202 (77.1%)	60 (22.9%)	
Bloating			
No	386 (73.1%)	142 (26.9%)	0.645
Yes	68 (70.8%)	28 (29.2%)	
Loss of appetite			
No	404 (72.1%)	156 (27.9%)	0.309
Yes	50 (78.1%)	14 (21.9%)	
Family history of gastric disease			
No	344 (73.5%)	124 (26.5%)	0.467
Yes	110 (70.5%)	46 (29.5%)	
Frequency of GI healthcare visits			
Rarely	224 (92.6%)	18 (7.4%)	< 0.001
Once a year	180 (57.3%)	134 (42.7%)	
2–3 times/year	32 (64.0%)	18 (36.0%)	
More than 3/year	18 (100.0%)	0 (0.0%)	
Type of Surgery			
Cholecystectomy	362 (79.7%)	92 (20.3%)	< 0.001
Gastric surgery	92 (54.1%)	78 (45.9%)	

### Age and biliary gastritis

**Fig. 2:**
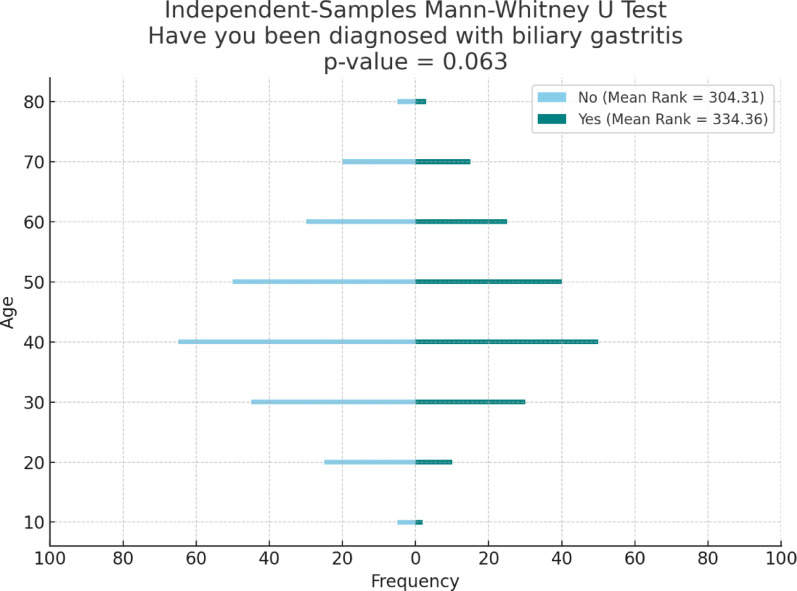
Association between age and biliary gastritis

The association between age and biliary gastritis diagnosis is illustrated in Fig. 2. Although the mean rank age was higher among participants with biliary gastritis, the difference did not reach statistical significance in unadjusted analysis (Mann–Whitney U test, *p* = 0.063).

In multivariable logistic regression analysis (Table 8), increasing age was independently associated with lower odds of biliary gastritis (adjusted OR = 0.95 per year, 95% CI 0.93–0.98), while smoking was strongly associated with higher odds (adjusted OR = 11.20, 95% CI 4.07–30.88). Regular physical activity and higher fruit and vegetable intake were independently associated with lower odds of biliary gastritis. Higher caffeine intake and medication use were also inversely associated in adjusted analysis. The final model demonstrated acceptable explanatory power (Nagelkerke R² = 0.338) and classification accuracy (77.9%).

**Table 8:** Multivariable logistic regression analysis of factors associated with biliary gastritis (*N* = 624)

Variable	Adjusted OR (Exp[B])	95% CI	*p*-value
Age (years)	0.95	0.93–0.98	< 0.001
Female sex	0.69	0.37–1.29	0.246
Married	0.70	0.21–2.32	0.554
Unemployed	0.90	0.54–1.49	0.680
Smoking (yes)	**11.20**	**4.07–30.88**	**< 0.001**
Physical activity (1–2×/week)	**0.37**	**0.19–0.71**	**0.003**
Spicy food consumption	1.15	0.48–2.74	0.754
Fast food consumption	1.79	0.76–4.24	0.184
Fruit & vegetable intake (higher vs. low)	**0.29**	**0.15–0.55**	**< 0.001**
Caffeine intake (higher)	**0.48**	**0.24–0.97**	**0.041**
Gastric surgery (vs. cholecystectomy)	1.13	0.67–1.90	0.647
Medication use	**0.03**	**0.01–0.10**	**< 0.001**

Bold = statistically significant (*p* < 0.05)

Omnibus test: χ² = 165.96, *p* < 0.001 . Nagelkerke R² = 0.338 .Overall classification accuracy: 77.9%

Although the Hosmer–Lemeshow test suggested reduced calibration (p < 0.001), this finding is commonly observed in models with strong predictors and does not invalidate the regression analysis

## Discussion

The present study estimated the prevalence of biliary gastritis among adults with available diagnostic data and examined associated demographic, lifestyle, dietary, and clinical factors in the Kurdistan Region of Iraq. Biliary gastritis was identified in 26.7% of participants, indicating a substantial regional disease burden comparable to reports from other settings and underscoring the importance of region-specific epidemiological data [13, 14].

In bivariate analyses, female sex was associated with a higher prevalence of biliary gastritis, consistent with previous reports suggesting greater susceptibility among women, potentially related to hormonal influences on bile composition and gastric motility [3]. However, this association did not remain significant after multivariable adjustment, suggesting confounding by related factors. Marital status was significantly associated with biliary gastritis in bivariate analysis, with higher prevalence among married participants. However, this finding is likely confounded by age, as married individuals in this study were generally older and may have had longer cumulative exposure to dietary and clinical risk factors. Similarly, the absence of diagnosed cases among students should be interpreted cautiously. Students in this sample represented a younger subgroup with potentially lower exposure duration and reduced likelihood of undergoing diagnostic procedures such as endoscopy. In addition, the relatively small number of students limits statistical stability. These findings therefore likely reflect differences in age distribution and healthcare utilization rather than true protective or risk effects of marital or occupational status.

Unemployment was associated with a higher prevalence of biliary gastritis, a finding that contrasts with studies reporting increased gastritis prevalence among individuals exposed to prolonged working hours [15]. This discrepancy underscores the context-specific nature of socioeconomic influences on gastrointestinal health. In the Kurdistan Region, lower socioeconomic status may be linked to poorer dietary quality, delayed healthcare utilization, and increased burden of untreated gastrointestinal symptoms, which may contribute to higher observed prevalence [16].

Dietary factors demonstrated consistent associations with biliary gastritis. Fast food consumption was associated with higher prevalence, supporting existing evidence that diets rich in fat and low in fiber may promote bile stasis, impaired gastric emptying, and mucosal injury [11]. Conversely, higher fruit and vegetable intake was inversely associated with biliary gastritis, aligning with prior studies suggesting that fiber-rich diets may reduce duodenogastric reflux and bile-induced gastric injury [17].

Caffeine intake showed a more complex pattern. In bivariate analysis, higher caffeine intake was associated with biliary gastritis, whereas multivariable analysis demonstrated an inverse association after adjustment. This apparent inconsistency may reflect confounding or reverse causation, whereby individuals experiencing gastrointestinal symptoms modify caffeine consumption following medical advice or symptom onset. Accordingly, the observed inverse association should not be interpreted as a protective biological effect, but rather as a behavior-related association inherent to cross-sectional designs [18, 19].

Several clinical conditions were significantly associated with biliary gastritis. Gallstone disease demonstrated a strong association, consistent with established evidence that biliary pathology can exacerbate duodenogastric reflux and subsequent gastric mucosal injury [3, 20]. Liver disease was also associated with biliary gastritis, likely reflecting altered bile composition and impaired bile flow, which may increase gastric exposure to cytotoxic bile acids [21]. Medication use was inversely associated with biliary gastritis in the adjusted analysis; however, medication use likely acts as a proxy for healthcare engagement or symptom management rather than a true protective biological factor.

In contrast, diabetes mellitus was not significantly associated with biliary gastritis, differing from some previous reports [22]. This discrepancy may reflect regional variations in diabetes management, lifestyle patterns, or metabolic profiles, highlighting the importance of local context in interpreting epidemiological findings [23].

Gastrointestinal symptoms, particularly nausea, vomiting, and abdominal pain, were significantly associated with biliary gastritis, consistent with the recognized clinical manifestations of bile-induced gastric irritation [7]. Heartburn and bloating were not associated, supporting the distinction between bile reflux–related gastritis and acid-predominant gastroesophageal reflux disease [24].

Healthcare utilization patterns were also relevant. Participants reporting annual or more frequent gastrointestinal healthcare visits exhibited higher prevalence of biliary gastritis, likely reflecting increased symptom burden and diagnostic opportunity rather than increased disease incidence. This finding underscores the influence of healthcare access and utilization on observed prevalence, particularly in regions with limited diagnostic resources [25].

Surgical history was associated with biliary gastritis in unadjusted analysis, with higher prevalence observed among participants who had undergone gastric surgery compared with those who had undergone cholecystectomy alone. However, this association did not persist after multivariable adjustment, suggesting that the observed relationship may be confounded by other clinical or lifestyle factors. This observation aligns with previous evidence that postoperative anatomical alterations may impair pyloric function and promote duodenogastric reflux [26].

This study contributes novel region-specific evidence on biliary gastritis from the Kurdistan Region of Iraq, where epidemiological data remain scarce. Unlike large longitudinal studies conducted in high-resource settings, the present study reflects real-world clinical practice in a context characterized by variable access to diagnostic endoscopy and differences in healthcare-seeking behavior. These contextual factors may influence both disease detection and observed associations. Additionally, dietary patterns in this population, including relatively low fruit and vegetable intake and increasing fast-food consumption—may differ from those reported in other populations, potentially modifying risk profiles. While longitudinal studies provide stronger evidence on temporal relationships, the current cross-sectional design offers valuable baseline data and highlights context-specific determinants that are essential for guiding local public health strategies. Nevertheless, the findings should be interpreted as associations rather than causal effects, and further prospective studies are warranted. These findings provide important baseline evidence for the Kurdistan Region, where comparable epidemiological data remains limited.

This study has several strengths, including a relatively large sample size and inclusion of both urban and rural populations, enhancing generalizability within the region. Use of a structured questionnaire and partial verification through medical records strengthens internal consistency.

### Limitations

The cross-sectional design precludes causal inference and limits temporal interpretation of associations. Several exposures and symptoms were self-reported, introducing potential recall bias. Diagnosis of biliary gastritis relied on existing clinical documentation or prior endoscopy, which may have led to underestimation of true prevalence due to limited diagnostic access. Furthermore, as the study was facility-based and included only individuals who sought healthcare services, the findings may not be fully generalizable to the broader community, particularly those who did not access healthcare or undergo diagnostic evaluation. Finally, genetic factors were not assessed and should be explored in future studies, particularly given emerging evidence of gene–environment interactions in biliary and reflux-related disorders [27, 28].

## Conclusion

Biliary gastritis is common among adults with available diagnostic data in the Kurdistan Region of Iraq and is associated with unfavorable dietary patterns, biliary pathology, and gastrointestinal symptoms. These region-specific findings underscore the importance of targeted prevention strategies and improved access to diagnostic endoscopy, particularly in high-risk and underserved populations. Further longitudinal research is recommended to clarify temporal relationships and support evidence-based interventions.

## Data Availability

The datasets used and/or analysed during the current study available from the corresponding author on reasonable request.
